# Optical molecular imaging and theranostics in neurological diseases based on aggregation-induced emission luminogens

**DOI:** 10.1007/s00259-022-05894-7

**Published:** 2022-07-04

**Authors:** Peili Cen, Youyou Zhou, Chunyi Cui, Yen Wei, Zhen Cheng, Shuizhu Wu, Hong Zhang, Mei Tian

**Affiliations:** 1 Department of Nuclear Medicine and PET Center, The Second Affiliated Hospital of Zhejiang University School of Medicine, Hangzhou, 31009 Zhejiang China; 2 Institute of Nuclear Medicine and Molecular Imaging of Zhejiang University, Hangzhou, 31009 Zhejiang China; 3 Key Laboratory of Medical Molecular Imaging of Zhejiang Province, Hangzhou, 31009 Zhejiang China; 4 Department of Chemistry and the Tsinghua Center for Frontier Polymer Research, Tsinghua University, Beijing, 100084 China; 5 Molecular Imaging Center, Shanghai Institute of Materia Medica, Chinese Academy of Sciences, Shanghai, 201203 China; 6 State Key Laboratory of Luminescent Materials and Devices, Guangdong Provincial Key Laboratory of Luminescence From Molecular Aggregates, College of Materials Science and Engineering, South China University of Technology, Wushan Road 381, Guangzhou, 510640 China; 7 College of Biomedical Engineering & Instrument Science, Zhejiang University, Hangzhou, 310014 Zhejiang China; 8 Key Laboratory for Biomedical Engineering of Ministry of Education, Zhejiang University, Hangzhou, 310014 Zhejiang China

**Keywords:** Aggregation-induced emission, Fluorescence imaging, Theranostics, Brain vasculature, Neurological diseases

## Abstract

Optical molecular imaging and image-guided theranostics benefit from special and specific imaging agents, for which aggregation-induced emission luminogens (AIEgens) have been regarded as good candidates in many biomedical applications. They display a large Stokes shift, high quantum yield, good biocompatibility, and resistance to photobleaching. Neurological diseases are becoming a substantial burden on individuals and society that affect over 50 million people worldwide. It is urgently needed to explore in more detail the brain structure and function, learn more about pathological processes of neurological diseases, and develop more efficient approaches for theranostics. Many AIEgens have been successfully designed, synthesized, and further applied for molecular imaging and image-guided theranostics in neurological diseases such as cerebrovascular disease, neurodegenerative disease, and brain tumor, which help us understand more about the pathophysiological state of brain through noninvasive optical imaging approaches. Herein, we focus on representative AIEgens investigated on brain vasculature imaging and theranostics in neurological diseases including cerebrovascular disease, neurodegenerative disease, and brain tumor. Considering different imaging modalities and various therapeutic functions, AIEgens have great potential to broaden neurological research and meet urgent needs in clinical practice. It will be inspiring to develop more practical and versatile AIEgens as molecular imaging agents for preclinical and clinical use on neurological diseases.

## Introduction

Molecular imaging is a revolutionizing approach to studying the inner mechanisms, helping diagnosis of diseases, designing new drugs, and assessing the efficacy of therapies, which makes it possible to visualize complex biochemical processes involved in pathophysiological states, in real time, in different aspects from living cells, tissues, organs, to intact subjects [1–3]. And it has been widely developed and investigated in biomedical applications based on special and specific imaging agents in neuroscience [4], oncology [5, 6], cardiology [7], gene therapy [8], cell tracking [9], and theranostics [10]. The imaging agents attracted much attention to researchers and were designed and synthesized based on various structures such as small molecules, peptides, aptamers, engineered proteins, and nanoparticles for single-, dual-, and multiple-imaging modalities both in vitro and in vivo [1, 11–14]. As one kind of them, organic fluorogens have been commonly accepted as outstanding optical imaging agents for molecular imaging, but always limited by notorious aggregation-caused quenching (ACQ) [15]. Recently, a unique phenomenon named aggregation-induced emission (AIE) was discovered by Tang’s group in 2001, which showed completely opposite to ACQ characteristics of conventional organic fluorogens in the concentrated state due to intramolecular π-π stacking [16]. The mechanism of AIE is the restriction of intramolecular motion (RIM) including restriction of intramolecular rotation (RIR) and restriction of intramolecular vibration (RIV) [17]. When the AIE luminogens (AIEgens) are in the solid state or aggregated, they emit intense fluorescence signals, and nearly no emission can be detected in the diluted solution. In fact, AIE processes have been reported to be also associated with other intramolecular processes such as J-aggregate formation (JAF), twisted intramolecular charge transfer (TICT), and excited-state intramolecular proton transfer (ESIPT) [18, 19]. AIEgens not only inherit advantages from conventional organic fluorogens like simple operations, high-fluorescence quantum yield, and good biocompatibility, but also exhibit better photostability and stronger emission with a large Stokes shift, which help to resist photobleaching and have a good potential for biomedical investigations in the complicated environment [20, 21]. Furthermore, AIEgens are endowed with excellent specific and sensitive targeting and therapeutic ability based on the molecular structure and optical characteristics to achieve image-guided theranostics, which can exert phototherapy including photodynamic therapy (PDT) and photothermal therapy (PTT) in the targeted sites [22–26]. In comparison with single therapy provided by AIEgens themselves, fabrication of AIEgens with drugs, peptides, or other structures can achieve more efficient treatment by combining phototherapy from AIEgens and other therapies from modified parts [27, 28]. The finding of the AIE phenomenon and corresponding development of AIEgens has expanded available agents for optical molecular imaging and image-guided theranostics in biomedical research.

As the most essential part of the nervous system, the brain participated in all pathophysiological processes of the human body. The basic structure of the brain is the neurovascular unit (NVU) that contains neurons, microglia, astrocytes, vascular endothelial cells, and pericytes, and plays a fundamental function to control blood–brain barrier (BBB) permeability and cerebral blood flow, and maintains the balance of microenvironment [29, 30]. Normal physiological processes of the brain need brain vasculature to deliver nutrition, signaling molecules, and metabolic wastes that combine brain and other organs and tissues [31–33]. And the endothelial cells of vasculature are stubborn components of the BBB to protect delicate brain parenchyma against complicated environments outside of the brain and maintain normal structures, precise functions, and numerous biological processes, such as angiogenesis [34], vascular leakage [35], and leukocyte extravasation [36, 37]. When neurological diseases occur either structurally or functionally, they always suggest huge disasters for the individual that bring damages, injure life quality, and become a hard burden for families and societies. It is becoming a substantial burden on individuals and society that affect over 50 million people worldwide [38]. Common neurological diseases such as cerebrovascular disease [39], neurodegenerative disease [40], and brain tumor [41], have been continuously bothering humans for a long time and still demand suitable approaches for theranostics [42, 43]. To investigate theranostics for neurological diseases, many noninvasive imaging modalities have been developed for different aspects, such as X-ray, ultrasound, computed tomography (CT), magnetic resonance imaging (MRI), single photon emission computed tomography (SPECT), and positron emission tomography (PET) [44–46], for which spatiotemporal resolution is still a limitation that obstructs our learning about neurological diseases. Conventional fluorescence imaging has excellent spatiotemporal resolution but is easily influenced by autofluorescence of other tissues and limited by penetration when excitation and emission wavelengths are in the range of visible light. It is more obviously interfered with when investigated on brain imaging and image-guided theranostics through the intact scalp and skull covering brain [47, 48]. To overcome this shortcoming, AIEgens have been explored and designed with excitation by multi-photon like two-photon fluorescence imaging [49] and three-photon fluorescence imaging [50] or at near-infrared (NIR) windows (700–1700 nm)[51], of which perform deep penetration, low autofluorescence, low scattering and high signal-to-noise ratio (SNR) to help brain imaging. Therefore, they are becoming efficient approaches for molecular imaging and image-guided theranostics in neurological diseases. Considering the obvious advantages of AIEgens, they are good candidates for optical imaging agents to explore the pathophysiological processes of neurological diseases through molecular imaging and image-guided theranostics [52].

**Fig. 1:**
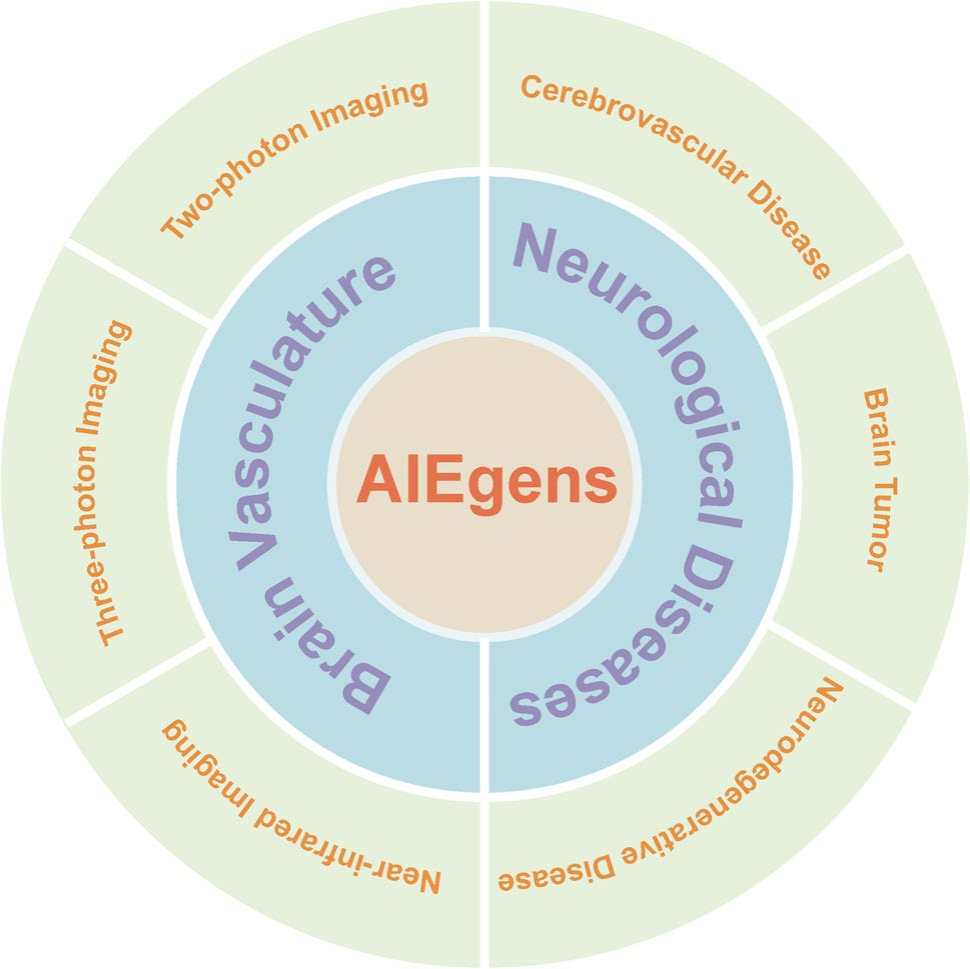
Schematic illustration of roles of AIEgens for optical molecular imaging and theranostics in neurological diseases

In this review, we present the recent advances in molecular imaging and image-guided theranostics in neurological diseases based on AIEgens (Fig. 1). We focused only on the representative AIEgens with clearly characterized properties and high-quality efficacy of imaging. First, we introduce and divide AIEgens for brain vasculature imaging into three parts based on imaging modalities—two-photon imaging, three-photon imaging, and NIR imaging—and then describe their formations, characters, and performances in biomedical applications. Second, we clarify and summarize AIEgens with various functions investigated in different neurological diseases from the perspectives of clinical needs, including cerebrovascular disease, neurodegenerative disease, and brain tumor. Finally, we conclude the advances of AIEgens in molecular imaging and image-guided theranostics in neurological diseases and give a new insight to promote the development of molecular imaging with AIEgens, with the great potential for preclinical/clinical translation.

## Brain vasculature imaging

Benefiting from large Stokes shift, strong fluorescence signal, outstanding photostability, high quantum yield, and good biocompatibility, AIEgens are regarded as ideal candidates for in vivo optical molecular imaging of brain vasculature. According to the different approaches to fluorescence imaging, these AIEgens were divided into three parts: two-photon fluorescence imaging, three-photon fluorescence imaging, and NIR fluorescence imaging. Then they were summarized with excitation, collection, largest imaging depth, and highest resolution in the biomedical applications (Table 1).

**Table 1:** Brain vasculature imaging based on AIE-based agents

Agents	Excitation (nm)	Collection (nm)	Largest imaging depth (μm)	Highest resolution (μm)	Operation	Ref
BTPEBT dots	800 (2PF)	515–569	424	-	Cranial window	Ref. [53]
TTF dots	800 (2PF)	-	300	-	Cranial window	Ref. [58]
TTS dots	900 (2PF)	-	350	4	Cranial window	Ref. [56]
BT dots	1040 (2PF)	590 longer	700	-	Cranial window	Ref. [60]
Azabenzanthrone derivates NPs	1040 (2PF)	560–700	280		-	Ref. [61]
TBP-b-TPA NPs	1040 (2PF)	800 shorter	700	1.55	Cranial window	Ref. [54]
AIETP NPs	1040 (2PF)	-	800	1.92	Cranial window	Ref. [57]
BTPETQ dots	1200 (2PF)	660–750	924	1.2	Cranial window	Ref. [59]
AIE dots	1300 (2PF)	810	1065	3.4	Cranial window	Ref. [55]
DCCN	1040 (2PF)	-	500	-	Cranial window	Ref. [59]
	1560 (3PF)	-	250	-		
DCDPP-2TPA NPs	1550 (3PF)	590 longer	300	2.4	Intact skull	Ref. [68]
TPATCN NPs	1550 (3PF)	590 longer	875	-	Cranial window	Ref. [65]
TPEPT NPs	1550 (3PF)	590–1035	505	-	Cranial window	Ref. [64]
TPATCN-NIR755 NPs	1550 (3PF)	780 longer	730	-	Cranial window	Ref. [63]
DCzPDI-NPs	1550 (3PF)	590–1035	450	2.31	Intact skull	Ref. [69]
BTF dots	1550 (3PF)	590 longer	400	0.95	Intact skull	Ref. [67]
TTF NPs	1560 (3PF)	590 longer	550	-	Cranial window	Ref. [66]
L897 NPs	808	1000 longer	1300 (relative to skin)	-	Intact skull and scalp	Ref. [70]
L1013 NPs	808	1000 longer	-	33.5	Intact skull and scalp	Ref. [72]
XA1 NPs	808	1250 longer	1300 (relative to skin)	-	Intact skull and scalp	Ref. [71]
DTPA-TBZ dots	808	1000 longer	-	100	Intact skull	Ref. [75]
P3c Pdots	808	1250 longer 1319 longer	-	-	Intact skull and scalp	Ref. [114]
OPTA-BTT dots	793	1100 longer	870	2.4	Cranial window	Ref. [74]
			700	5.2	Thinned skull	
TT3-oCB NPs	793	1500 longer	-	3.3	Intact skull	Ref. [76]
TQ-BPN dots	635	785–900	800	18.4	Cranial window	Ref. [77]

*2PF* two-photon fluorescence imaging, *3PF* three-photon fluorescence imaging

### Two-photon fluorescence imaging

As a common approach to generating a high-energy fluorescence signal in the visible region from low-energy irradiation in the NIR region, two-photon fluorescence imaging provides a unique and clear optical window for in vivo imaging, which has advantages of deep-tissue penetration, low autofluorescence, and low phototoxicity. Numerous AIEgens have been investigated for two-photon fluorescence imaging on brain vasculature [53–62]. They were all designed with a high two-photon absorption cross section and large quantum yield to contribute to deep penetration and high resolution.

**Fig. 2:**
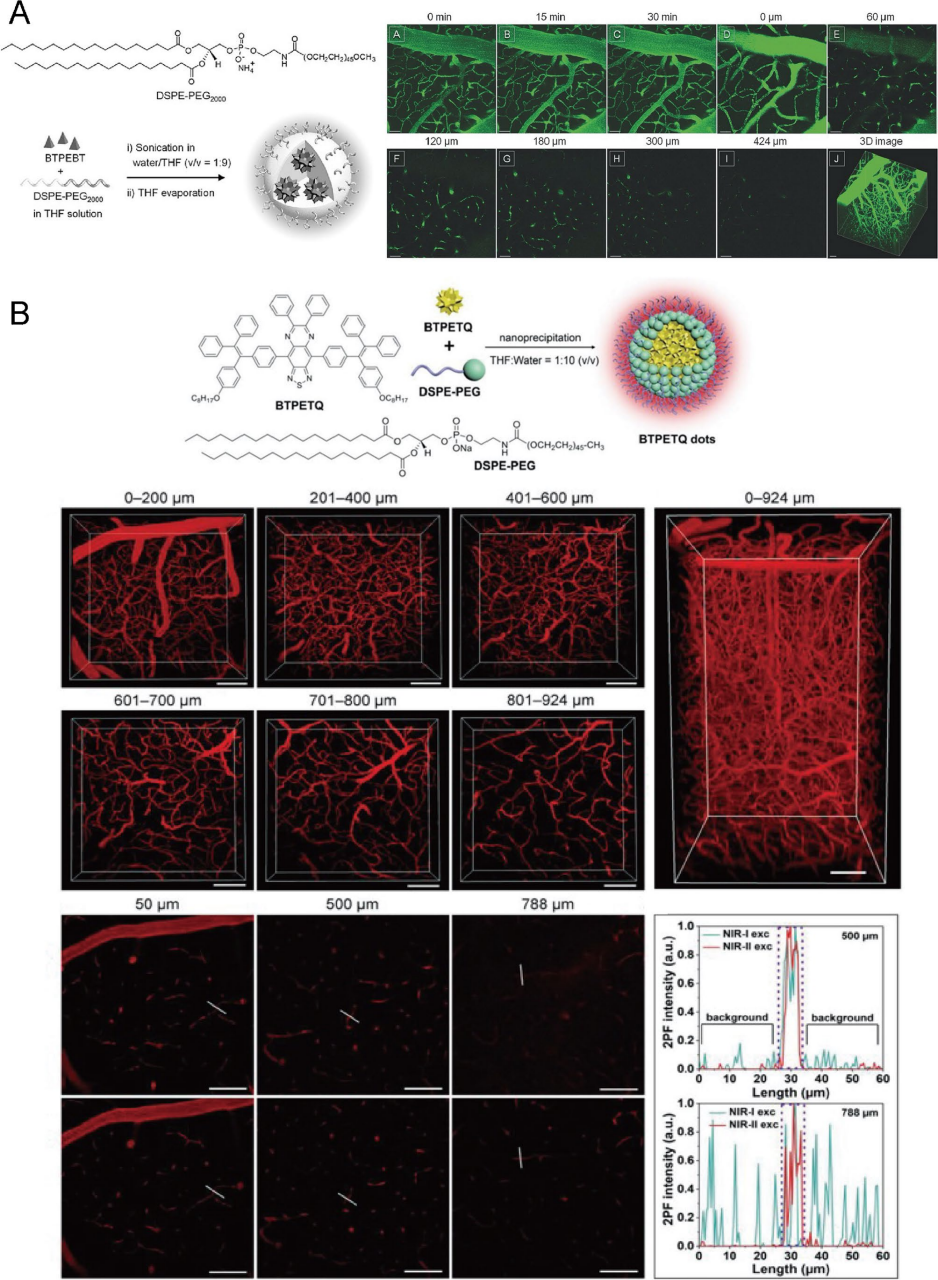
Two-photon fluorescence imaging of brain vasculature through the cranial window on mice based on AIEgens. **A** Molecular structure and synthetic route of BTPEBT dots, time lapse (0–30 min), and different depths (0–424 μm) imaging for 3D reconstruction of mouse brain vasculature through two-photon fluorescence imaging. Adapted permission from Ref. [53]. Copyright © 2013 WILEY‐VCH Verlag GmbH & Co. KGaA, Weinheim. **B** Molecular structure, synthetic route of BTPETQ dots, imaging of different depths (0–924 μm) through a two-photon microscope, and 3D reconstruction of mouse brain vasculature and visualization of brain capillaries with good spatial resolution comparing NIR-I and NIR-II at 500 μm and 788 μm. Adapted permission from Ref. [59]. Copyright © 2019 WILEY‐VCH Verlag GmbH & Co. KGaA, Weinheim

The first AIEgen for two-photon imaging explored to visualize brain vasculature was BTPEBT (Fig. 2A) [53]. It was composed of two TPE structures as the donor and one 2,1,3-benzothiadiazole as the acceptor which helped the TICT effect. And the corresponding AIE dots were formed by encapsulation with DSPE-PEG_2000_, and the AIE dots showed absorption peaks at both 318 and 425 nm and an emission peak at 547 nm. The BTPEBT dots had a large two-photon absorption cross Sect. (10.2 × 10^4^ GM at 810 nm) and a high quantum yield (62 ± 1%), which had a potential for in vivo visualization. In brain vasculature imaging through a cranial window under a two-photon microscope excited by an 800-nm laser, major vessels could be visualized clearly, as well as smaller capillaries. Deeper than 400 μm of microvasculature could be detected with high resolution. Furthermore, the brain vessels could be imaged by BTPEBT dots over 30 min with a continuous bright fluorescent signal. Other AIE dots with different structures such as TTF dots [58], TTS dots [56], BT dots [60], 11-azabenzanthrone derivates NPs [61], TBP-b-TPA NPs [54], AIETP NPs [57] have been also reported to image brain vasculature successfully with high resolution and deep penetration through two-photon fluorescence imaging, benefiting from their good optical characters.

Although BTPEBT dots can realize around 400 µm-depth visualization of brain vasculature, deeper penetration and higher resolution are still urgently needed to achieve the whole-brain vasculature visualization. From the recent advances, BTPETQ dots [59] were representative AIEgens designed to improve spatial resolution, which visualized brain blood vessels as small as around 1.2 μm of Gaussian full width at half maximum (FWHM), one of the smallest values achieved by AIEgen-assisted two-photon fluorescence imaging in vivo of brain vasculature (Fig. 2B). After fabricating by encapsulating BTPETQ molecules with DSPE-PEG_2000_ through nanoprecipitation, the dots showed an emission peak at 700 nm, a large two-photon absorption cross section of 7.63 × 10^4^ GM at 1200 nm, and a high quantum yield of 19 ± 1%. After being injected retro-orbitally, brain vessels of mice were imaged through a cranial window by the dots excited by 920 nm and 1200 nm lasers, respectively. Brain blood vessels were clearly visualized with the maximal depth reaching 924 μm excited at 1200 nm, which was deeper than NIR-I (920 nm) excitation. And the high spatial resolution of BTPETQ dots was around 1.2 μm detected at the depth of 900 μm.

Another approach to improve image quality is deepening the penetration in brain vasculature imaging. TQ-BPN nanodots [55] were reported to achieve among the largest detection depths of in vivo two-photon fluorescence imaging, as well as high spatial resolution. TQ-BPN was a crab-shaped AIEgen composed of several twisting phenyl/naphthyl rotators and a planar core structure to afford both high fluorescence quantum yield and efficient two-photon activity of 1.22 × 10^3^ GM. The encapsulated AIE dots were prepared with TQ-BPN and Pluronic F-127. In intravital two-photon fluorescence imaging, excitation at 1300 nm at the NIR-II region helped to reconstruct 3D vasculature of mouse brain with an excellent spatial resolution (~ 3.5 μm) at deep regions of white matter and the hippocampus at more than 960 μm. It could also detect tiny blood vessels of nearly 5 μm at the depth of 1065 μm, which was one of the deepest penetrations in brain vasculature imaging by two-photon fluorescence in vivo.

Many other AIEgens were also performed for brain vasculature imaging through a two-photon microscope with deep penetration, high spatial resolution, high signal-to-background ratio (SBR), and 3D brain vasculature reconstruction. However, in two-photon fluorescence imaging, it always needs invasive operations to open the skull to form a cranial window or skull-thinning techniques which injure animals and limit clinical translation.

### Three-photon fluorescence imaging

Three-photon fluorescence imaging further decreases autofluorescence and phototoxicity and increases tissue penetration and spatial resolution, in comparison with two-photon fluorescence imaging.

**Fig. 3:**
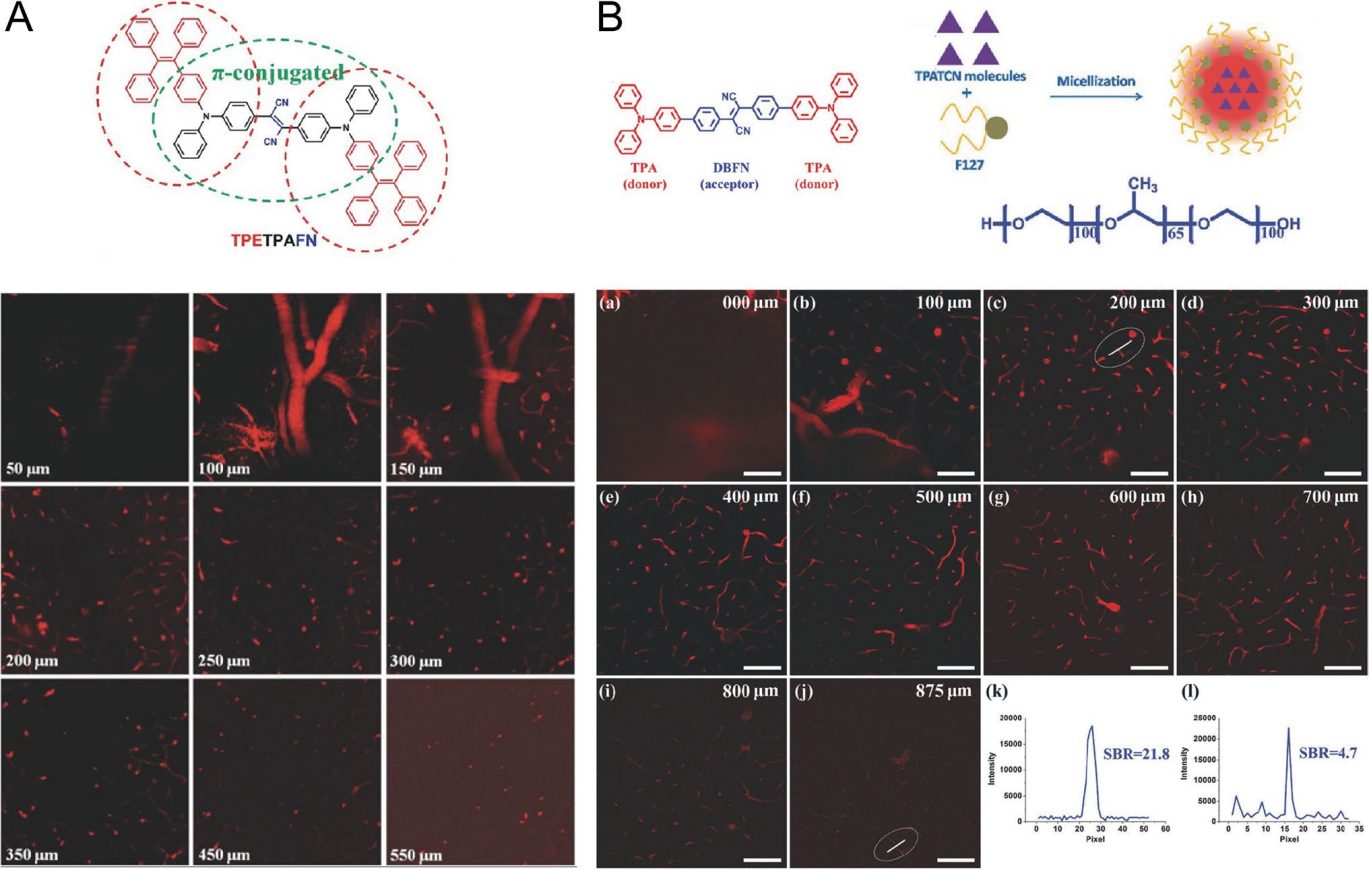
Three-photon fluorescence imaging of brain vasculature through the cranial window on mice based on AIEgens. **A** Molecular structure of TTF (TPETPAFN) and the corresponding NPs on different depths (0–550 μm) imaging of mouse brain vasculature through three-photon fluorescence imaging. Adapted permission from Ref. [66] Copyright © 2015 WILEY‐VCH Verlag GmbH & Co. KGaA, Weinheim. **B** Molecular structure and synthetic route of TPATCN NPs and mouse brain vasculature imaging as deep as 875 μm with good SBR by three-photon microscope. Adapted permission from Ref. [65]. Copyright © 2017 WILEY‐VCH Verlag GmbH & Co. KGaA, Weinheim

It has been reported that AIEgens could image brain vasculature by three-photon fluorescence imaging through craniotomy with high penetration and resolution [62–66], which had similar efficacy compared with those by two-photon fluorescence imaging mentioned above. TTF was the first one reported to image brain vasculature through three-photon fluorescence imaging (Fig. 3A) [66]. It had the typical donor-π-acceptor-π-donor structure, which contributed to enhancing multiphoton absorption and high-order three-photon-excited luminescence excited by a 1560-nm femtosecond laser. Then, TTF was applied to achieve 3PL brain imaging of mice after being encapsulated with DSPE-mPEG to form nanoparticles. In vivo 3PFI of mouse brain vasculature, the AIE dots were injected intravenously to uncover the vascular architecture of the brain at various vertical depths and the largest imaging depth in the mouse brain was achieved at 550 μm. TPATCN [65] was another representative example, and it had triphenylamine (TPA) as the donor and diphenylfumaronitrile (DBFN) as the acceptor to form a donor–acceptor-donor structure, which endowed it with a narrow band gap (Fig. 3B). The NIR emission character was attributed to the strong light absorption of TPA and the high-fluorescence efficiency of DBFN. After being encapsulated with F127 to form nanoparticles, the dots were used to build a vivid 3D reconstruction of the brain vasculature with the penetration depth of 875 μm under the excitation of a 1550-nm fs laser. Based on the TPATCN structure, another AIEgen was further developed by using TPATCN as the donor and NIR775, a NIR dye, as the acceptor, which showed fluorescence resonance energy transfer (FRET) characteristics as high as 90%. The encapsulated TPATCN-NIR755 NPs [63] had an emission peak at 785 nm and could also reconstruct mouse brain vasculature as deep as 730 μm. Moreover, other AIEgens reported including DCCN [62], TPEPT NPs [64], and TTF NPs [66], have all been confirmed with the ability of three-photon fluorescence imaging on brain vasculature through cranial windows with deep penetration and high resolution.

**Fig. 4:**
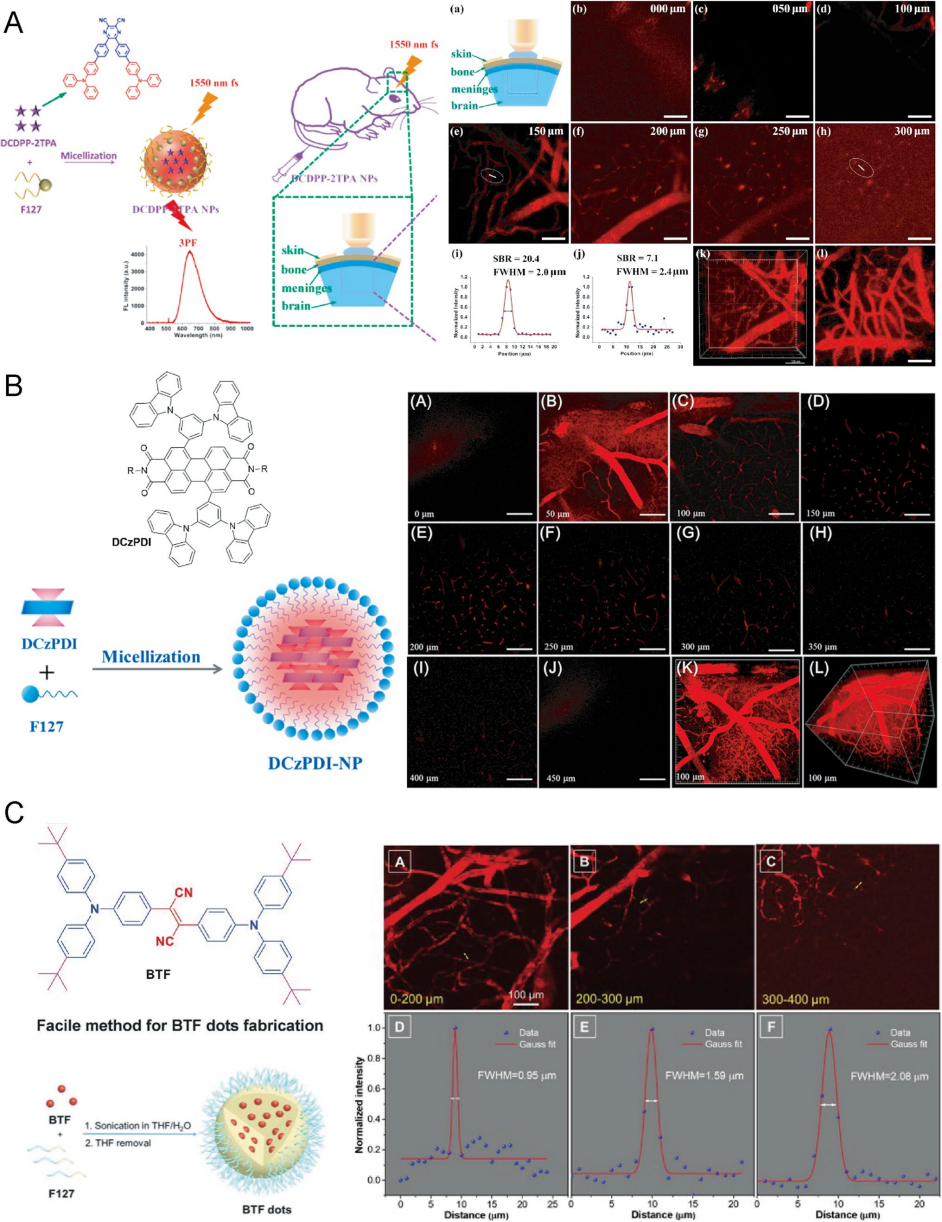
Three-photon fluorescence imaging of brain vasculature without cranial window on mice based on AIEgens. **A** Molecular structure of DCDPP-2TPA, synthetic route, and emission spectrum of the corresponding NPs, and schematic illustration on mouse brain vasculature visualization at 0–300 μm with the intact skull which helped recognize 2.0 μm capillaries with 20.4 SBR. Adapted permission from Ref. [68]. Copyright © 2017, American Chemical Society. **B** Molecular structure of DCzPDI, synthetic route of the corresponding NPs, and 450 μm-deep vasculature 3D reconstruction of mouse brain without the intact skull. Adapted permission from Ref. [69]. Copyright © 2018, American Chemical Society. **C** Molecular structure of BTF, synthetic route of BTF dots, and nearly 0.95 μm spatial resolution of vessels in mouse brain vasculature without cranial window. Adapted permission from Ref. [67]. Copyright © 2020 WILEY‐VCH Verlag GmbH & Co. KGaA, Weinheim

More importantly, some AIEgens for three-photon imaging can visualize directly through the intact skull without invasive operations to minimize injuries and pain to the organism [63–69]. To achieve brain vasculature imaging without craniotomy and skull-thinning operations, a deep-red emissive AIEgen (DCDPP-2TPA) [68] was synthesized and encapsulated with Pluronic F-127 to form nanoparticles for three-photon fluorescence imaging of mouse brain vessels (Fig. 4A). DCDPP-2TPA NPs had a three-photon absorption cross section of 2.95 × 10^−79^ cm^6^ s^2^, which was larger than other reported organic dyes. And the NPs showed very high photostability because of very little decrease in 3PF intensity after continuous scanning for a long time. As for in vivo mouse brain vasculature imaging, DCDPP-2TPA NPs could detect vessels as deep as 785 μm and distinguish capillaries as small as 2.4 μm at the depth of 300 μm with good SNR.

DCzPDI-NPs [69] were reported to achieve the largest penetration (450 μm) by three-photon imaging on brain vasculature through the intact skull (Fig. 4B). DCzPDI contained perylene diimide (PDI) and 1,3-di(9H-carbazol-9-yl)benzene, in which an enlarged size of the latter was used as the isolation to converse oppositely from ACQ to AIE characteristics by decreasing π-π stacking. And in the aggregated state, DCzPDI exhibited an emission peak at 638 nm in the deep-red window with a quantum yield of 12.3%. After using F127 for fabrication by nanoprecipitation, DCzPDI NPs showed a three-photon cross section of 6.8 × 10^−80^ cm^6^s^2^ under a 1550-nm laser. For brain vasculature imaging in vivo, the brain blood vessels at different depths could be acquired, and the tiny capillaries could be observed with clear structures in the depth from 150 to 450 μm. And the spatial resolution was measured to be 1.26 μm at the 150-μm depth, and 2.39 μm at the 435-μm depth, which benefited from clear observation of the tiny blood vessels.

BTF dots [67] achieved another kind of improvement in three-photon fluorescence imaging on brain vasculature, which got the best spatial resolution of 0.95 μm through the intact skull (Fig. 4C). BTF had TPA carrying tert-butyl (t-Bu) groups as the strong donor and fumaronitrile (FN) moiety as the acceptor, which showed efficient emission at the far-red/near-infrared (FR/NIR) region, a high quantum efficiency of 36.1%, and a large three-photon absorption cross section of 2.56 × 10^−79^ cm^6^ s^2^ at 1550 nm when formulated into AIE nanodots. The corresponding dots displayed an absorption peak at 500 nm and an emission peak at 645 nm which extended to the NIR region. For in vivo visualization of blood vessels in mouse brain with the intact skull, the diameter of the tiny capillary was detected as 0.95, 1.59, and 2.08 μm at the depths of 200, 300, and 400 μm, which demonstrated the potential for in vivo deep-tissue imaging, especially the brain. In addition, the BTF dots were also the first of adopting AIE dots to visualize the cerebral thrombosis process crossing the intact skull on a mouse model with high penetration and good image quality.

### Near-infrared fluorescence imaging

Near-infrared (NIR) light can exhibit deep penetration in biological tissues, and fluorescence imaging in the near-infrared windows has the advantages of low tissue scattering and nearly no background autofluorescence, guaranteeing that it is a promising imaging modality in noninvasive visualization of deep tissues such as the brain without invasive operations like three-photon fluorescence imaging.

**Fig. 5:**
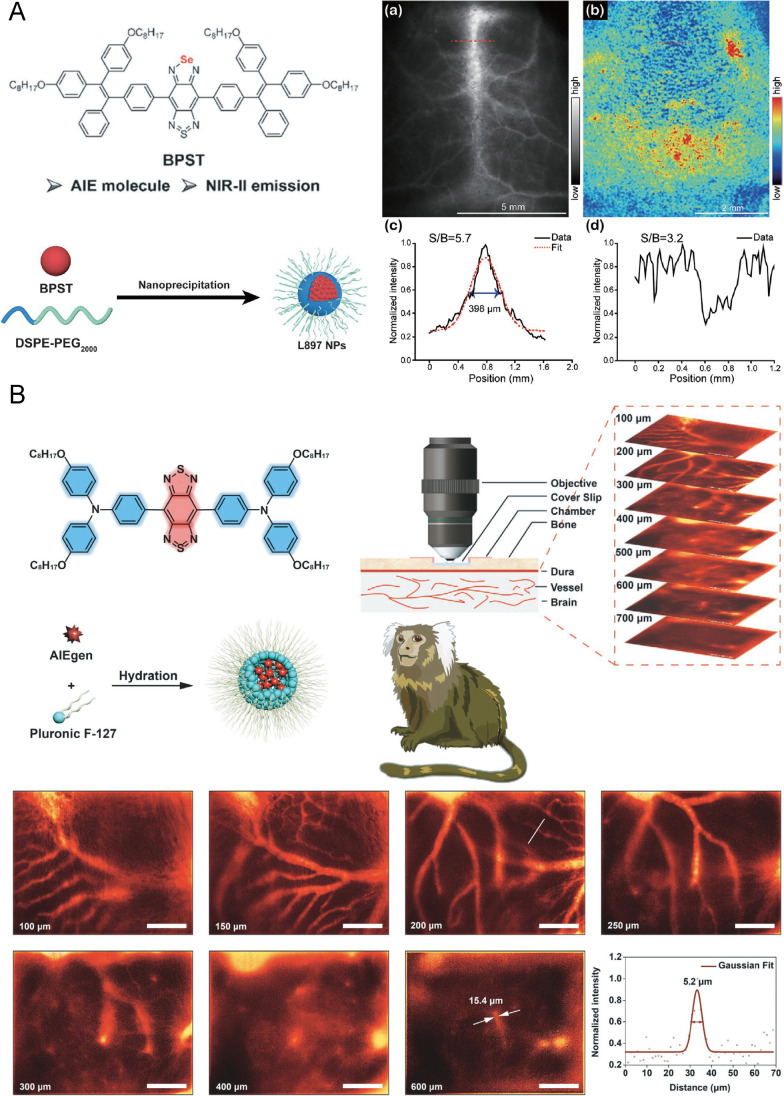
Near-infrared imaging of brain vasculature without cranial window on rodents and nonhuman primates. **A** Molecular structure of BPST with AIE and NIR-II emission, synthetic route of L897 NPs based on BPST, and brain vessel imaging as deep as 1300 μm from the intact scalp and skull on mice. Adapted permission from Ref. [70]. Copyright © 2019 WILEY‐VCH Verlag GmbH & Co. KGaA, Weinheim. **B** Molecular structure of OPTA-BTT and synthetic route of the corresponding dots, and brain vasculature visualization of thinned-skull marmoset with depth as 700 μm and detection of tiny capillaries as small as 5.2 μm. Adapted permission from Ref. [74]. Copyright © 2021 WILEY‐VCH Verlag GmbH & Co. KGaA, Weinheim

An AIEgen with the emission peak at 897 nm and an emission tail at the NIR-II window named BPST was adopted for in vivo imaging of brain blood vessels after being encapsulated into nanoparticles with DSPE-PEG_2000_ (named L897 NPs) [70] (Fig. 5A). The resultant L897 NPs had two absorption bands that centered at 347 and 711 nm and displayed a quantum yield of 5.8%. When applied on the mouse model, the L897 NPs were excited at 808 nm and the signal was collected in the NIR-II region, and the images of cerebral vasculature were detected as deep as 1.3 mm from the skin surface. The mouse brain vessels were visualized clearly by L897 NPs with a SBR as 5.7 through the intact skull and scalp. The similar penetration depth realized by L897 NPs in mouse brain blood vessel imaging was also achieved by NIR imaging based on XA1 NPs [71] excited at 808 nm and collection at the wavelength longer than 1000 nm.

The maximum spatial resolution in NIR imaging of brain vasculature through the intact skull and scalp was 33.5 μm, which was reported in the application of L1013 NPs [72]. The L1013 NPs were formed by encapsulating BTPPA using nanoprecipitation method. The NPs exhibited an emission peak at 1013 nm and an emission tail extending to 1400 nm in the NIR-II window under an 808-nm laser excitation. And the NPs had a quantum yield of 9.9%. In the imaging of mouse cerebral vessels, cerebral vessels could be clearly and sharply visualized through the intact scalp and skull by a low power density and short exposure, including inferior cerebral veins, superior sagittal sinus, and transverse sinus. The L1013 NP-based imaging had a good SBR ratio of 6.56, and the smallest diameter of vessels was measured as 33.5 μm.

A great improvement in clinical translation from the rodents to the primates was that the AIE dots used for brain vasculature imaging were applied on the nonhuman primates [73]. It was reported that OTPA-BBT dots [74] could visualize brain vasculature with an excretable NIR-II AIEgen with a large molar absorption coefficient of 5 × 10^4^ M^−1^ cm^−1^ at 770 nm (Fig. 5B). After being encapsulated into organic nanoparticles using F127, OTPA-BTT dots showed an emission peak at 1020 nm and an extremely high quantum yield of 13.6%, and an ultrabright luminescence emitted beyond 1100 nm and even beyond 1500 nm at the NIR-IIb window. In the cerebrovascular imaging on marmosets with a high spatial resolution through the thinned skull, the imaging depth below the thinned skull could reach nearly 700 μm and the capillary of 5.2 μm was distinctly identified at 200 μm. Meanwhile, the OTPA-BTT dots could also help to monitor cortical blood flow as functional imaging with high temporal resolution through the thinned skull of marmosets. The blockage of cortical vasculatures below the thinned skull was visualized in a real time, in which the blood flow arrest or even reflux in the other side branches could be observed.

Other AIEgens like DTPA-TBZ dots [75], P3c Pdots, and TT3-oCB NPs [76] also had the ability of NIR imaging on brain vasculature with intact skull and scalp, while TQ-BPN dots [77] must make a cranial window to image brain vasculature as deep as 800 μm and visualize nearly 18.4 μm small vessels.

## Image-guided theranostics in neurological diseases

### Cerebrovascular disease

Normal cerebrovascular structures play essential roles in environmental balance in the brain in vivo. When the normal structures had dysfunctional changes, cerebrovascular disease occurs and damages brain structure and function [39].

**Fig. 6:**
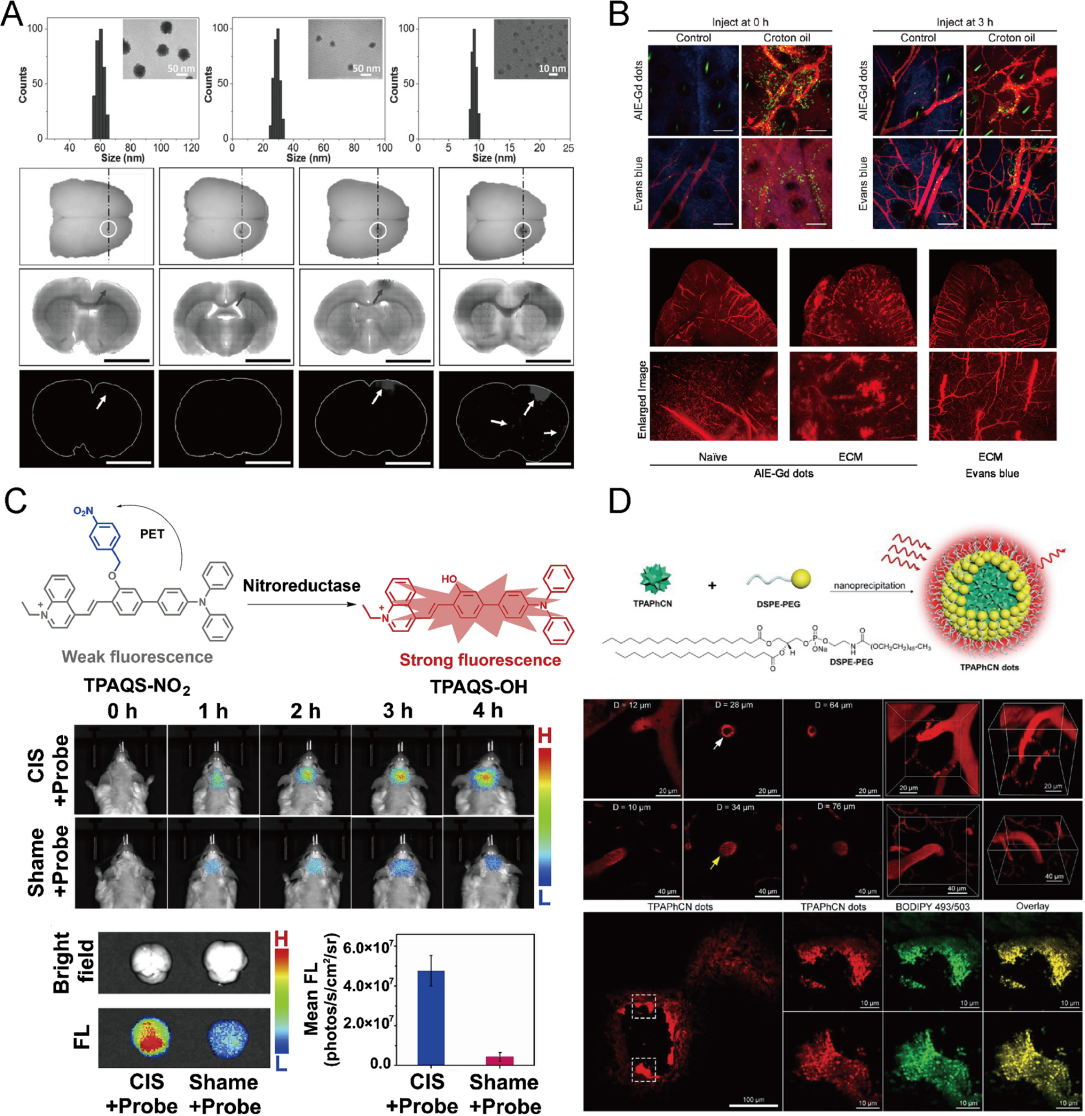
Optical molecular imaging of pathological changes in cerebrovascular disease based on AIEgens. **A** The sensitive detection of BBB leakage and damage on mouse models with suitable size of AIE NPs between 10, 30, and 60 nm based on TPETPAFN. Adapted permission from Ref. [79]. Copyright © 2016 WILEY‐VCH Verlag GmbH & Co. KGaA, Weinheim. **B** BBB damage and microbleeding imaging with AIE-Gd dots compared with Evans Blue at 3 h after injection. Adapted permission from Ref. [81]. Copyright © 2017 Published by Elsevier Ltd. **C** Molecular structure and turn-on by nitroreductase of TPAQS-NO_2_ and image hypoxia environment in brain on mouse models with cerebral ischemic stroke in vivo and ex vivo. Adapted permission from Ref. [84]. Copyright © 2020 Elsevier B.V. D) Synthetic route of TPAPhCN dots and visualization of atherosclerosis plaques in brain vessels. Adapted permission from Ref. [86]. Copyright © 2021 WILEY‐VCH Verlag GmbH & Co. KGaA, Weinheim

The leakage of BBB is one kind of pathological process in cerebrovascular disease [78]. Detecting BBB leakage as early as possible can help early diagnosis and therapies to avoid harmful factors which may injure neural cells. TPETPAFN [79] was designed with AIE characteristics and used to form nanoparticles of the suitable size to detect BBB leakage at the accurate phase of ischemic stroke in the photothrombotic ischemia rat model (Fig. 6A). In comparison with traditional Evans Blue (EB) [80], a commonly used contrast agent for assessing BBB damage, TPETPAFN NPs exhibited more sensitivity and less toxicity, which guaranteed the potential for preclinical and clinical usage. In all different sizes of the NPs, 60 nm NPs showed the highest brightness but failed to pass through BBB. Ten-nanometer NPs could cross BBB but showed the lowest fluorescence and lacked sensitivity. Successfully, 30-nm NPs were observed to be the most sensitive and selective probe for assessing BBB damage, indicating the importance of size to design BBB probes. Furthermore, TPETPAFN was used by another group to produce AIE-Gd nanodots by encapsulated with lipid-PEG and then coupled with gadolinium, which could be applied for detecting BBB leakage and the bleeding of microvasculature [81] (Fig. 6B). In normal mouse vessels, no significant leakage of AIE-Gd dots from the vasculature was observed as well as no increase in background signal in the interstitium, even for smaller capillaries in the soft meninges. In ECM-infected mouse vessels, AIE-Gd dots leaked into the interstitium around the bleeding microvasculature and formed punctate aggregates, whereas EB did not. AIE-Gd nanodots could also realize quantitative determination of vascular leakage and accumulation in tissue using destructive ICP-MS.

Hypoxia is induced by obstruction of brain vessels and reduction of blood flow, which forms a common pathological feature of ischemic in cerebrovascular disease [82]. In the hypoxic microenvironment, nitroreductase (NTR) is always overexpressed, regarded as a sensor that reflects the degree of hypoxia [83]. TPAQS-NO_2_ was designed as an activable probe for hypoxia detection by responding to NTR, which was composed of an electron acceptor quinolinium and an electron donor triphenylamine moiety [84] (Fig. 6C). TPAQS-NO_2_ was highly selective for NTR and showed a large Stokes shift (186 nm) when activated, which could effectively avoid interference caused by molecular self-absorption. When TPAQS-NO_2_ was applied on a mouse model of cerebral ischemia, a significant fluorescence signal was observed clearly in the brain region as early as 1 h after injection and increasing in 4 h, while the sham-operated group showed a much weaker fluorescence signal.

Atherosclerotic plaques not only appear in carotid arteries but also are formed in brain vasculature, becoming one of cerebrovascular diseases [85]. AIE dots were designed to overcome strong hydrophobicity that restricts usages of the current lipid-specific luminogens for in vivo detection [86] (Fig. 6D). The organic dots with AIE characteristics could label and image lipid-rich tissues of atherosclerotic plaques in brain vasculature, as well as in fatty liver and carotid arteries. The dots could keep stable in the aqueous solution with good specificity of targeting lipids and strong three-photon fluorescence in the FR/NIR region excited by a NIR-II laser, which contributed to efficient labeling and imaging of lipids in the deep tissues in vivo.

In addition to the diagnosis of abnormal changes in brain and related cerebrovascular diseases, monitoring processes of cellular therapies are used to rescue the damages of cerebrovascular diseases especially ischemic stroke [87]. Stem cell transplantation is very popular for researchers and has become an emerging therapeutic approach for ischemic stroke treatment which has a great potential for clinical translation [88, 89]. Some reports have already imaged and monitored stem cells by AIEgens in ischemic stroke treatment, to investigate the mechanism of stem cell therapy [90, 91]. The AIE molecule mentioned above, TPETPAFN [90], was also reported to form AIE-NPs to track and monitor mouse neural progenitor cells and hESC-derived neurons in cultured neurons. The NPs showed a high degree of penetration into cells and presented intracellular long-term retention in vitro without altering the neuronal proliferation, differentiation, and viability. And using AIE-NPs to label neuronal grafts could be monitored in the mouse brain striatum at various time points post-transplantation for at least 1 month. Other AIE NPs encapsulating different AIE cores were developed to track another type of stem cell, bone marrow stromal cells (BMSCs). BMSCs are commonly used for stem cell therapy and show promising therapeutic outcomes for stroke treatment, in which the fate of BMSCs is still not clear. And TPEEP [91] was designed with AIE and NIR emission characteristics for tracking BMSCs in the whole process of stroke treatment and assessing the therapeutic effects, which helped to improve the success rate. After being fabricated into NPs, the obtained NPs showed excellent tracking performance of BMSCs in vitro and in vivo. Furthermore, the NP-labeled BMSCs were observed to migrate to the stroke lesion site to yield bright red fluorescence on a rat photothrombotic ischemia model. And their good biocompatibility in vivo was confirmed by immunofluorescence staining that the NP labeling did not affect the normal function of BMSCs.

### Brain tumor

Brain tumor is the most prevalent devastating disease in the brain which seriously harms human health because of its invasive growth in the central nervous system and causing discernible neurological symptoms rapidly with an extremely poor prognosis [92]. Glioma is the most common primary brain tumor, which still met difficulties to distinguish tumor margins for precise surgery and deliver drugs efficiently crossing BBB, especially glioblastoma [93, 94].

**Fig. 7:**
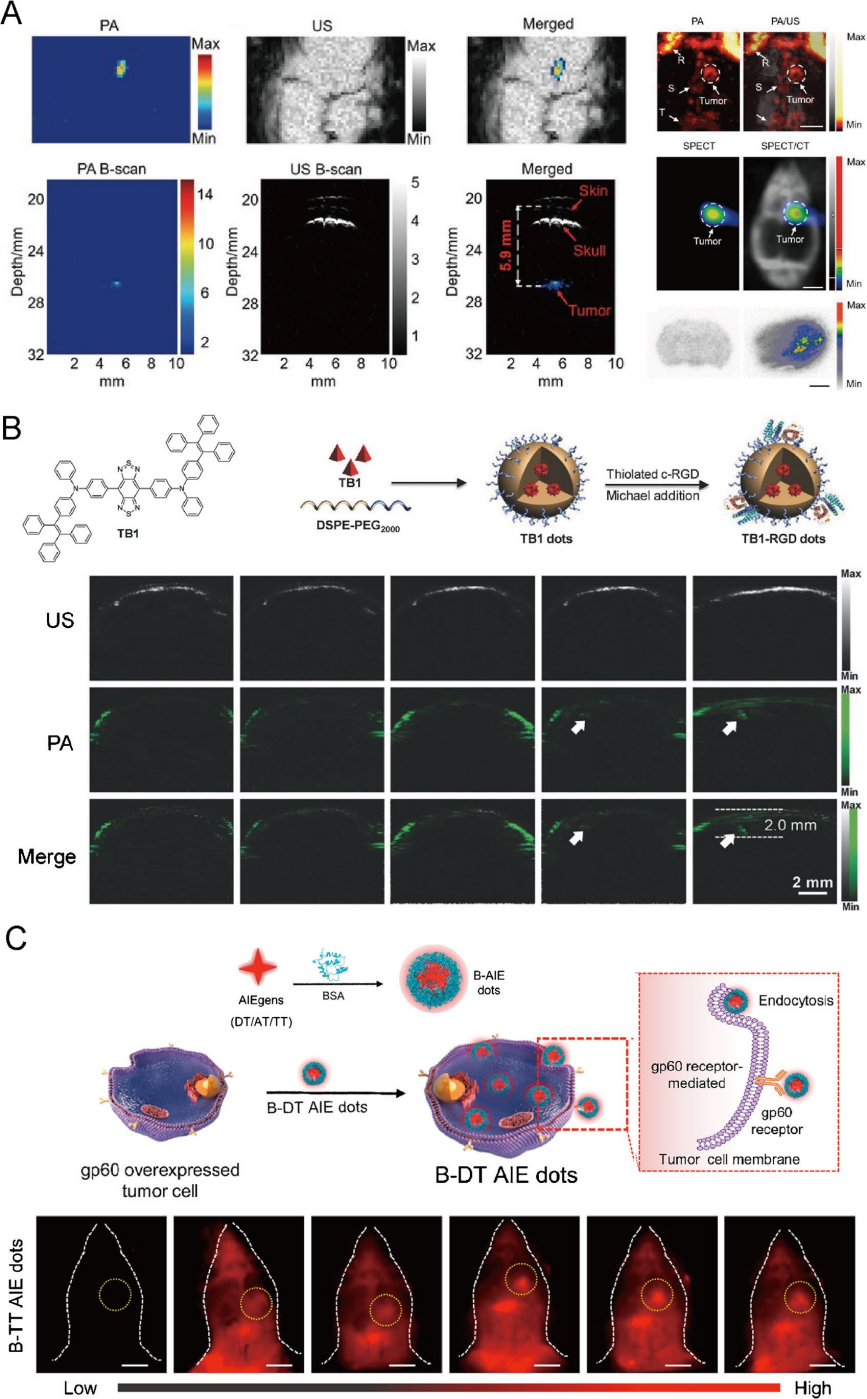
Optical molecular imaging for brain tumor visualization and delineation. **A** Photoacoustic imaging (PAI) by A1094@RGD-HBc for brain gliomas imaging in vivo, consistent with SPECT/CT. Adapted permission from Ref. [95]. Copyright © 2019 WILEY‐VCH Verlag GmbH & Co. KGaA, Weinheim. **B** Molecular structure of TB1, synthetic route of TB1-RGD dots, and PA imaging to visualize brain tumor. Adapted permission from Ref. [96]. Copyright © 2018 WILEY‐VCH Verlag GmbH & Co. KGaA, Weinheim. **C** Synthetic route of B-AIE dots (DT/AT/TT), illustrated endocytosis by gp60 overexpressed tumor cell and using B-TT AIE dots to achieve orthotopic glioma NIR-II imaging and image-guided surgery. Adapted permission from Ref. [97]. Copyright © 2022, American Chemical Society

Several AIEgens have been reported to help delineate brain gliomas margins for precise imaging and image-guided surgery. Mesoionic dye A1094 encapsulated in Arg-Gly-Asp-modified hepatitis B virus core protein (RGD-HBc) [95] was designed and synthesized for effective NIR-II photoacoustic imaging (PAI) of brain gliomas (Fig. 7A). After labeling A1094@RGD-HBc with ^131^I, enhanced PA signals in tumors could be observed with great SBR, which was proved by ultrasensitive SPECT imaging of gliomas. Other AIE dots based on TB1 molecules [96] were also reported to have potential for in situ brain tumor imaging for precise diagnosis through dual fluorescent imaging and PAI (Fig. 7B). The TB1-RGD dots could cross BBB and accumulate in tumors in 24 h with good SBR (4.4) in NIR-II fluorescence imaging. Furthermore, strong NIR-I PA signals could be detected and reached the maxima at 24 h post-injection in the tumor through intact scalp and skull, and the tumor region could be clearly visualized as deep as 2.0 mm. To enhance BBB penetration and tumor accumulation, consolidating albumin to AIEgens to form nanoprobes is effective in induced endocytosis to cross BBB. An albumin-based AIE nanoprobe, B-TT AIE dots [97] could induce endocytosis mediated by the gp60 receptor on orthotopic glioma and achieve in vivo NIR-II imaging and image-guided tumor surgery in mouse models (Fig. 7C). Conjugated polymer NPs (CP NPs) named PBT NPs [98] were also reported for brain tumor imaging. The NPs were proven with dual-modality brain imaging in the NIR-II window and successfully enabled to mapping deep microscopic brain tumor of 2 mm under the intact skull and scalp through NIR-II PAI with the SBR of 7.2 after focused ultrasound-induced BBB opening. The ultrasmall (-8 nm) TQFP-10 NPs [99] with NIR-II fluorescence and long blood circulation time were designed for efficient orthotopic glioblastoma imaging which could distinguish tumor tissues efficiently from normal tissues in both subcutaneous and in situ glioblastoma.

**Fig. 8:**
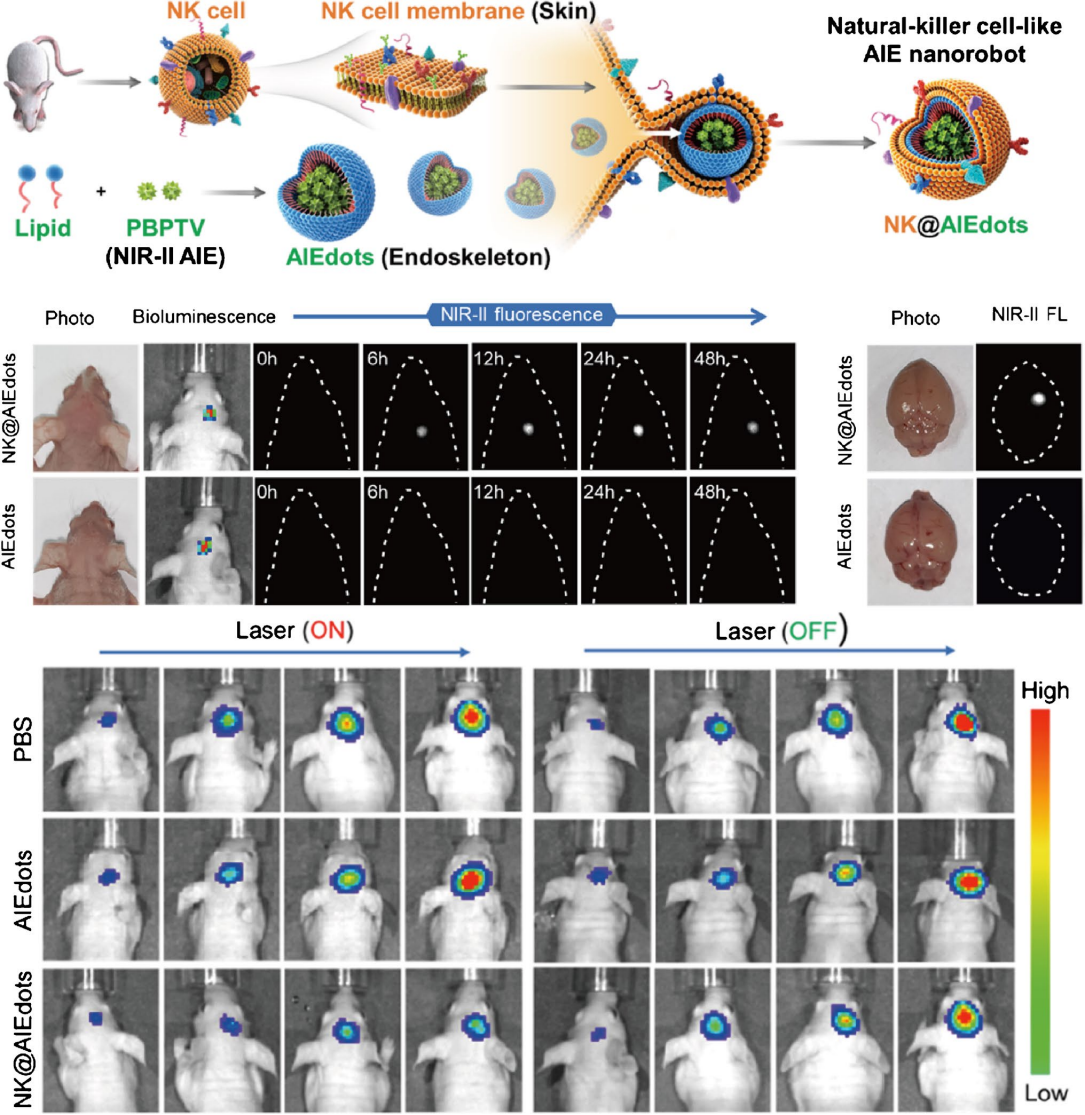
Synthetic route of NK@AIEdots by covering AIEdots as endoskeleton with NK cell membrane as skin to cross BBB and applied for NIR-II imaging of glioblastoma and efficiently suppress tumor growth by PTT in vivo. Adapted permission from Ref. [100]. Copyright © 2016 WILEY‐VCH Verlag GmbH & Co. KGaA, Weinheim

Precise imaging and delineation of brain tumors based on AIEgens provides a potential for tumor surgery. However, the complicated structure and function of brain always obstruct and limit the adoption of surgery to remove tumors in brain. Interestingly, the ability of AIEgens has been proven not only in imaging but also to bring new approaches to brain tumor therapies based on the structure of AIE molecules which are different from chemotherapy, radiotherapy, and immunotherapy, such as PDT and PTT. A representative case was that NK@AIEdots [100] were successfully used for glioblastoma with excellent NIR-II imaging and PTT abilities (Fig. 8). NK@AIEdots were designed as nanorobots by coating a natural kill cell membrane on a highly bright NIR-II AIE-active conjugated polymer (PBPTV), showing a high NIR-II quantum yield of ~ 7.9% in water and good biocompatibility. Benefiting from the NK coat to trigger an intracellular signaling cascade, they could cross the BBB silently by disrupting tight junction and the actin cytoskeleton and accumulate in glioblastoma with high-contrast and through-skull imaging. And under NIR light illumination, the tumor growth was obviously inhibited by NK@AIEdots. Other AIEgens like ApoE-Ph NPs [101], BK@AIE NPs [102], and FA-cRGD-TNSP NPs [103] were also confirmed to kill brain tumor cells in vivo through PTT with different pathways to cross BBB. ApoE-Ph NPs were reported with fluorescence imaging ability at 1550 nm and higher efficiency of PTT, which could cross BBB and target glioblastoma benefited from the ApoE structure. And bradykinin (BK) endowed BK@AIE NPs with selective penetration through the blood-tumor barrier (BTB) by activating adenosine receptors to enhance transportation and accumulation inside tumors. They realized NIR imaging and photothermal therapy (PTT) on glioblastoma and improved the survival rate of xenograft mice. Utilizing folate and cRGD peptide to modify, FA-cRGD-TNSP NPs could also exhibit superior ability to target glioblastoma cells in vitro and efficient accumulation on the margins and insides of tumors in vivo. And the NPs showed a great inhibition of glioblastoma progression through PTT. Furthermore, BK@AIE NPs also induced activation of natural killer cells, CD3( +) T cells, CD8( +) T cells, and M1 macrophages to enhance therapeutic efficacy for glioblastoma.

### Neurodegenerative disease

Neurodegenerative diseases have become a hard burden and influence nearly all families in the aging society nowadays. A common pathological change in neurodegenerative diseases is amyloid aggregation [104], which would form amyloid fibrils and plaques and always damage neurons in brain and influence normal brain function. Recently, AIEgens play an essential role in the amyloid aggregates detection, amyloid kinetics monitoring, and amyloid inhibitor development[105].

**Fig. 9:**
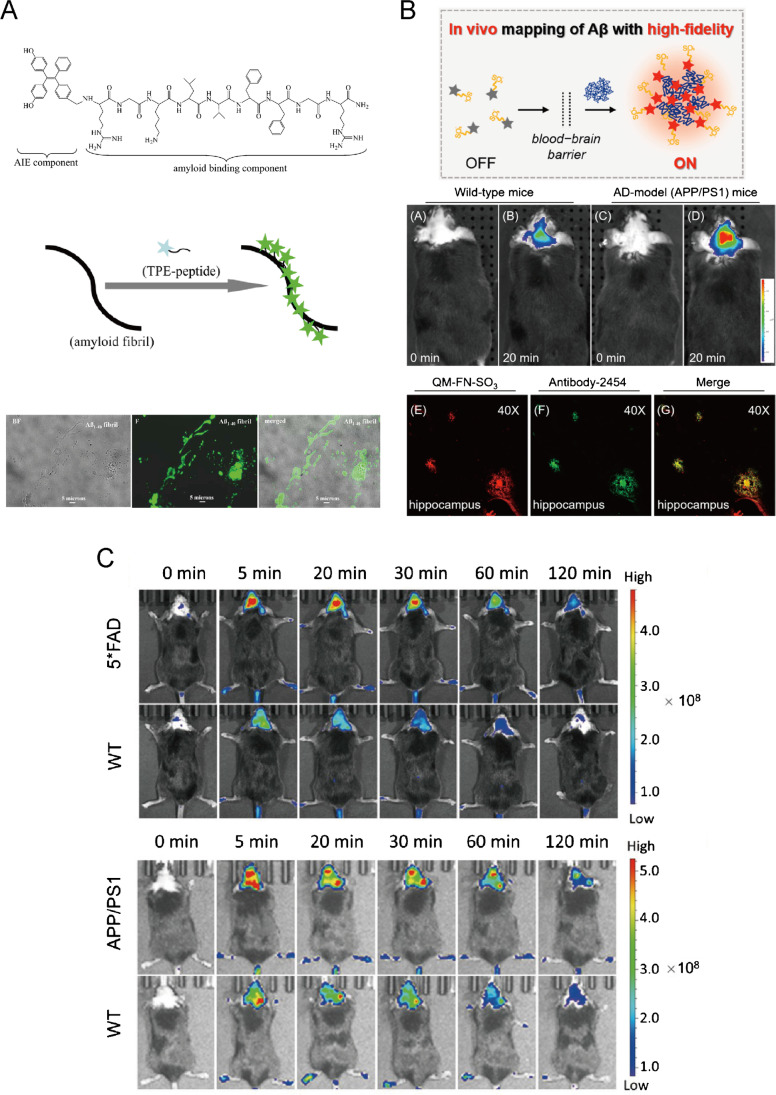
Amyloid fibrilization visualization by AIEgens in neurodegenerative disease. **A** Molecular structure of TPE conjugated with amyloid-like structures and detects Aβ fibril sensitively. Adapted permission from Ref. [106]. Copyright © 2015 American Chemical Society. **B** Illustrated principle of QM-FN-SO_3_ for in vivo mapping of Aβ with high-fidelity application for Aβ imaging at 20 min after injection on APP/PS1 mice as AD models in vivo and confirmed ex vivo with Antibody-2454 in the hippocampus. Adapted permission from Ref. [112]. Copyright © 2019, American Chemical Society. **C** In vivo visualization of Aβ deposits in real time for 120 min in a 5*FAD and APP/PS1 mouse model by AIE-CNPy-AD. Adapted permission from Ref. [113]. Copyright © 2021, Science China Press and Springer-Verlag GmbH Germany, part of Springer Nature

The direct imaging amyloid fibrils are adopting amyloid-like structures as targeting moieties, which endow AIEgens with specific imaging ability. Following the strategy above, an AIEgen-conjugated AIE structure and amyloid structure binding peptide was successfully synthesized and proven that could detect amyloid fibrils sensitively and monitor their dynamics in vitro [106], which was better than traditional thioflavin T (ThT) (Fig. 9A). It emitted green fluorescence in the presence of amyloid aggregation while no emission could be observed at the monomer state. Similar results were found in the investigations of TPE-TPP [107] and ASCP [108], which contained no amyloid-like structures as targeting moieties.

Not just detection, AIEgens were further used for screening drugs that could inhibit amyloid fibrils. AIE@amyloid [109] was composed of EPB and UAA-amyloid through click reaction, and then successfully applied for screening amyloid inhibitors against amyloid-β protein (Aβ) and α-synuclein (αSN), which had the potential as candidates for monitoring progression and therapeutic efficacy of Alzheimer’s disease (AD) and Parkinson’s disease (PD). Furthermore, it found tolcapone as an effective amyloid inhibitor from a large-scale database, which could inhibit both the aggregation and cytotoxicity of Aβ and αSN and further obviously improved the brain function of the Aβ mouse model.

Some reports directly detect Aβ fibrils as a sensitive sensor for AD diagnosis, as well as an inhibitor against the formation of Aβ fibrils. A supramolecular AIE glyconanoparticle (AIE-GNP) [110] was reported to detect Aβ peptides and fibrils sensitively, which was synthesized by the supramolecular assembly between fluorescent glycoprobes (DK1 and DK2) and a silole-based AIEgen (DES). And the monomeric Aβ peptides and their fibrils were detected in a ratiometric manner, determined to stem from the disruption of a FRET process between the closely coated glycoprobes and the AIE particle upon interaction with protein/peptide analytes. Cur-N-BF2 [111] was designed and confirmed with not only Aβ fibril and plaque detection but also inhibition of Aβ fibrillation, disassembly of Aβ fibrils, and protection of neuronal cells from Aβ fibrils in vitro. Moreover, QM-FN-SO_3_ [112] could further achieve Aβ plaque detection in vivo with remarking binding affinity and high-fidelity feedback (Fig. 9B). And AIE-CNPy-AD [113] was also successfully applied in vivo to detect Aβ in the mouse model (Fig. 9C). It was designed by integrating Aβ deposit-favored geometry, amphiphilic and zwitterionic molecular structure, extended D-π-A electronic structure, and 3D conformation into one molecule. Many advantages including high specificity, high affinity to Aβ deposits, bright red/NIR fluorescence, low interference from autofluorescence, high SNR, and high contrast brought AIE-CNPy-AD a great potential for in vivo visualization of Aβ deposits in real time as early as 4 months in the young adult mouse model.

## Conclusion and perspectives

Molecular imaging and image-guided theranostics based on luminogens with AIE characteristics have been developed and investigated widely in the past few decades. Compared with conventional commercial dyes, AIEgens displayed superior advantages such as large Stokes shift, high photostability, and high quantum yield, benefiting their biomedical applications with strong fluorescence signal, good biocompatibility, high signal-to-noise ratio, and good phototherapeutic efficacy. To meet the urgent demands of clinical practice on neurological diseases, AIEgens have been endowed with multi-photon excitation, NIR emission, specific targeting ability, and good therapeutic efficacy. In this review article, the recent advances in molecular imaging and image-guided theranostics in neurological diseases by AIEgens were summarized and concluded by some representative examples.

AIEgens with two-/three-photon excitation or NIR emission successfully visualized normal brain vessels and capillaries with deep penetration and high resolution, which reached nearly micrometer level of spatial resolution and helped a vivid 3D reconstruction of brain vasculature. In two-photon fluorescence imaging of brain vasculature, invasive operations like craniotomy to make a cranial window were necessary to improve imaging quality that directly excited the AIE molecules aggregated in the blood circulation of brain without crossing the skull. When using three-photon fluorescence imaging by AIEgens to visualize brain vasculature, cranial windows and skull-thinning operations were not prerequisites. Importantly, not only rodents but nonhuman primates could be imaged by AIEgens without invasive operations in NIR imaging of brain vasculature, confirming their potential for clinical practice.

Neurological diseases including cerebrovascular disease, neurodegenerative disease, and brain tumor were investigated here by AIEgens, which realized targeted imaging and image-guided theranostics. For cerebrovascular disease, several pathological features were utilized to design and develop as the targets of AIEgens, such as BBB leakage, hypoxia, and atherosclerotic plaques mentioned above, and the corresponding AIEgens’ good performances in detecting these pathological changes in vivo were proven. Furthermore, cellular therapies like stem cell transplantation were tracked and investigated by AIEgens in the treatment of ischemic stroke, one kind of cerebrovascular disease. For tumor theranostics, AIEgens were widely developed and successfully applied. However, the design of AIEgens was restricted by BBB for brain tumor-targeted imaging and theranostics. Here, AIEgens reported for brain tumor applications were adopting different strategies to enhance BBB penetration that conjugated with peptides or proteins like RGD, albumin, ApoE structure, and receptor ligands like bradykinin, covering NK cell membrane and controlling the ultrasmall size of NPs and external conditions like ultrasound-induced BBB opening. In addition to crossing BBB to image and delineate margins of brain tumors like glioma and glioblastoma, the therapeutic functions of AIEgens like PDT and PTT and corresponding combined therapies brought new approaches to brain tumors and improved therapeutic efficacy. For neurodegenerative disease, AIEgens play an important role mainly in the detection of amyloid fibrils, especially Aβ for AD. Compared with traditional ThT, a gold standard used for amyloid fibril detection, AIEgens adopting an amyloid-like structure as targeted moieties could sensitively detect and image the formation of amyloid fibrils and plaques in vitro and in vivo, while some others were reported to detect amyloid fibrils using different strategies without amyloid-like structure. The development of AIEgens for the neurodegenerative disease could also help to screen corresponding inhibitors that inhibit the formation of amyloid fibrils and extenuate symptoms, with great potential for clinical usage.

The further development of AIEgens for molecular imaging and image-guided theranostics will require researchers to consider simultaneously complicated structures and functions of normal and abnormal conditions in the living organism. Brain vasculature imaging by representative AIEgens through two-/three-photon fluorescence imaging and NIR fluorescence imaging were categorized and summarized with their performances in biomedical applications. Considering the current achievement of AIEgens that visualize brain vasculature, the ideal AIEgens for brain vasculature imaging will show deeper penetration to visualize the whole brain and higher spatial resolution to observe capillaries as clear as possible, as well as no toxicity and noninvasive operations that guarantee clinical translation and. Moreover, PAI is another rapidly emerging modality in biomedical research with the advantages of noncontact operation, high optical resolution, and deep penetration, attracting attention from interdisciplinary fields. Many agents with various structures and characteristics have been synthesized and investigated for PAI in biomedical studies and expanding clinical usages, especially in vascular imaging, musculoskeletal imaging, and tumor therapies [115–117]. However, it stills lacks inspiring report AIEgens for PAI in normal brain vasculature. Therefore, AIEgens for PAI will be an attractive orientation for future development in brain vasculature imaging. Besides, different imaging modalities like CT, MRI, and PET are conventional imaging techniques to visualize brain structure and function, which have their own unique advantages that provide various and detailed brain information from different aspects. Several AIEgens have been already endowed with additional PET imaging modality in biomedical studies [118–121]. Thus, multimodal imaging that combines fluorescent imaging and conventional imaging modalities in the design and synthesis will be another promising orientation to enrich the imaging functions of AIEgens in brain. When AIEgens are going to be applied on neurological diseases for theranostics, it is necessary to premeditate their unique features that can encourage design and improve the sensitivity and specificity of AIEgens in applications, especially in vivo. In neurological diseases, there are many changes in the pathophysiological process including expression of intracellular proteins, disconnection of intercellular communication, and disbalance of the extracellular microenvironment in brain, which is highly valuable to be considered as the targets to exert targeted imaging and therapeutic functions. For example, the PDT and PTT of AIEgens that are always used in cancer therapy might be adopted to ablate other pathological tissues like atherosclerotic plaques formed in brain vessels to provide a new approach and avoid invasive surgery. Fabricating AIEgens into nanoparticles with various drugs can make AIE-based agents multifunctional and expand their usage in different fields. For brain tumor theranostics, PDT and PTT from AIEgens can be combined with conventional chemotherapy and radiotherapy, and the integration of imaging and theranostics will be better to enhance the therapeutic efficacy. For neurodegenerative diseases, there are too few instances of AIEgens that successfully inhibit amyloid aggregation and clear amyloid fibrils in the brain. It still requires effort to develop more AIEgens with therapeutic functions for neurodegenerative diseases. Besides, many other neurological diseases such as epilepsy, brain inflammation, and depression are waiting for new strategies to provide new imaging modalities and theranostics that are developed based on AIEgens. Furthermore, the structure of AIEgens for molecular imaging and image-guided theranostics should not be limited by the reports; other new structures will be encouraged to display a good performance, even better. Finally, it is essential to develop AIEgens and keep the potential clinical practice in mind for the future, which urgently demands clinical translation and brings a new choice to patients and doctors.
